# Magnetically Actuated Manipulation and Its Applications for Cartilage Defects: Characteristics and Advanced Therapeutic Strategies

**DOI:** 10.3389/fcell.2020.00526

**Published:** 2020-06-30

**Authors:** Chi Zhang, You-Zhi Cai, Xiang-Jin Lin, Yue Wang

**Affiliations:** ^1^Center for Sport Medicine, The First Affiliated Hospital, Zhejiang University School of Medicine, Hangzhou, China; ^2^Li Dak Sum & Yip Yio Chin Center for Stem Cell and Regenerative Medicine, Zhejiang University School of Medicine, Hangzhou, China; ^3^Spine Lab, Department of Orthopedic Surgery, The First Affiliated Hospital, Zhejiang University School of Medicine, Hangzhou, China

**Keywords:** magnetically actuated manipulation, magnetic nanoparticles, magnetic seeding, magnetic scaffolds, magnetic cell sheets, cartilage regeneration

## Abstract

For the fact that articular cartilage is a highly organized and avascular tissue, cartilage defects are limited to spontaneously heal, which would subsequently progress to osteoarthritis. Many methods have been developed to enhance the ability for cartilage regeneration, among which magnetically actuated manipulation has attracted interests due to its biocompatibility and non-invasive manipulation. Magnetically actuated manipulation that can be achieved by introducing magnetic nanoparticles and magnetic field. This review summarizes the cutting-edge research on the chondrogenic enhancements via magnetically actuated manipulation, including cell labeling, cell targeting, cell assembly, magnetic seeding and tissue engineering strategies.

## Introduction

When articular cartilage is lost, it has limited self-regeneration potential due to its exquisite structural design and avascular nature, which would subsequently progress to osteoarthritis.

To date, various strategies have been developed to enhance cartilage regeneration, including microfracture, autologous chondrocyte implantation (ACI), and stem cell therapy (Zhang et al., 2016). These methods provide advantages of mimicking some features found in native cartilage tissue (Enea et al., 2015; Gobbi and Whyte, 2016), but the phenomenon of deterioration in cartilage quality was unavoidable (Agung et al., 2006). In addition, simply seeding a scaffold with MSCs or other cells cannot reach sufficient cell density and cell condensation, which impeding the tissue regeneration (Shimizu et al., 2006). With the development of the biotechnology, cell manipulation based on magnetic force, in other words, magnetically actuated cell manipulation (MAM), which can be achieved by the synergy between magnetic nanoparticles (MNPs) and magnetic field, has attracted increasing attention (Delyagina et al., 2011; Xia et al., 2018). In magnetic field, after introducing MNPs into cells or materials, magnetic force could direct cellular and matrix organization. Thus, the method confers several advantages, including remoting control ability, providing a sufficient cell density, promoting cell concentration and adhesion, which are essential for cell differentiation and tissue regeneration (Ochi et al., 2014). Furthermore, biomedical applications based on MAM would hold promises for promoting cartilage regeneration.

Here, we reviewed the state-of-art research of magnetically actuated manipulation for cartilage regeneration. First, we introduce the influence of MAM on the biological behaviors of cells, including cell labeling and the approaches that make the cells magnetically responsive. Then, the applications of MAM *in vitro*, *in vivo*, and in clinical trial were further discussed. Finally, current limitations and future perspectives on MAM were given.

## Effects of MAM on the Biological Behaviors of Cells

MAM is achieved by the synergy between magnetic response element and magnetic field. As a magnetic response element, MNPs [e.g., superparamagnetic iron oxide (SPIOs) and paramagnetic molecules] have been used for cell labeling (Bulte, 2009). MNPs were mainly localized in phagocytic vacuoles, which could stably incorporate within cells (Son et al., 2015; Goncalves et al., 2017). Thus, MNPs were randomly dispersed but not accumulated intracellularly (Bakhru et al., 2012), and internalized MNPs were cleared through excretion routes or utilized by endogenous iron metabolism (Charlton et al., 2016), which avoiding toxicity. It was indicated that below 30 ug/ml, MNPs did not impair cell viability or metabolic cell activity (van Buul et al., 2011). *In vivo* assays indicated the accumulation of MNPs in the liver and spleen, and found that the MNPs did not modify any of the toxicological parameters with the increase of MNPs dose (Sharifi et al., 2012; Li et al., 2013; Ruiz et al., 2016). Furthermore, Mejias et al. (2013) observed the long-term toxicity of MNPs *in vivo*, and indicated that MNP did not compromise mouse survival, although acute toxicity were found 30 days after administration in some mouse, these were transient. Besides, the stemness and differentiation potential of MSCs was also not affected after MNPs labeling (Mohanty et al., 2018). In addition, MNPs could accelerate cell cycle progression by regulating the expression of cell cycle protein (Huang et al., 2009), and increase cells growth by reducing intracellular H_2_O_2_ through intrinsic peroxidase-like activity (Zhang et al., 2015). Furthermore, internalized MNPs did not particularly influence reactive oxygen species formation (Goncalves et al., 2017), but nanoparticles-mediated reactive oxygen species could influence the activation of the motigen-actived protein kinase pathways, which was important for promoting cell differentiation (Son et al., 2011; Rauch et al., 2013). Wang et al. (2016) indicated that MNPs could activate MAPK signal pathway to enhance osteogenic or chondrogenic differentiation of MSCs. And Hu et al. (2018) also found that cells labeled with MNPs had greater angiogenic functions, which could enhance bone or cartilage regeneration.

As another component, magnetic fields could regulate the biological behaviors of cells, such as the morphology, proliferation and differentiation, which may be relate to magnetomechanical interactions and radical pair effects when magnetic fields interactions with the cells (Miyakoshi, 2005; Zhang et al., 2014). Besides, magnetic fields could uncage bioactive factors by the magnetic nanoswitch to temporally regulate stem cell adhesion, differentiation, and mechanosensing (Kang et al., 2018). High-frequency magnetic fields would inhibit chondrocyte proliferation and induce cell apoptosis (Hsieh et al., 2008). However, a low-frequency magnetic field could be able to control the direction of magnetic labeled MSCs (m-MSCs) with a certain number, and no adverse influence was observed on chondrogenesis (Jaberi et al., 2011; Fayol et al., 2013; Goncalves et al., 2017). Furthermore, magnetic fields can moderate mast cell-mediated inflammatory reactions and accelerate clearance of M2 macrophages that is associated with inflammation resolving (Santos et al., 2015, 2016), and cell adhesion molecules upregulated when m-MSCs exposed to a MF (Caplan, 2007; van Buul et al., 2011).

## Application of MAM *In Vitro* for Cartilage Regeneration

**FIGURE 1:**
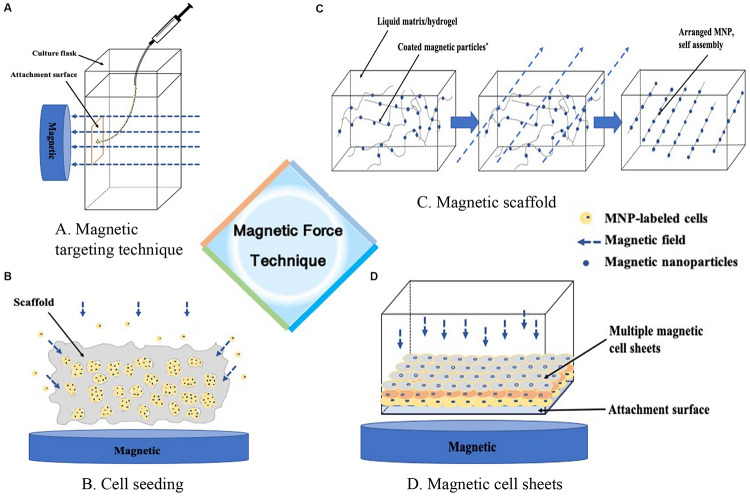
Magnetic force technique. **(A)** Guiding m-cells to a targeted site, **(B)** Enhancing the seeding efficacy of the cells into the scaffold, **(C)** Formation of ECM proteins assembly as building blocks in a liquid matrix or hydrogel, **(D)** Guiding cells into sheet-like structures to stack layer-by-layer, or containing different cocultured cell types.

**FIGURE 2:**
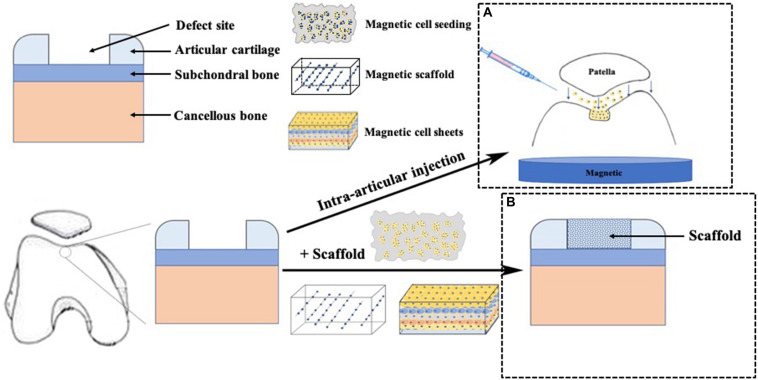
Application of magnetic force technique in cartilage repair. **(A)** cells labeled with MNPs were intra-articular injected and accumulated to the targeted cartilage defect sites using an external magnetic force, **(B)** A scaffold was placed in the cartilage defect site.

In general, we identified four technique notes explaining MAM, including cell targeting, magnetic seeding, magnetic scaffolds, cell assembly and other tissue engineering strategies. Table 1 and Figures 1, 2 briefly summarized the strategies of magnetically actuated manipulation.

**TABLE 1:** The procedures and characteristics of MAM.

**Technique**	**Procedure**	**Characteristics**
MTD	M-cells were intra-articular injected and accumulated to the targeted defect sites using magnetic force	Avoid excessive number of MSCs; less invasive and easy to handle; cover the defect site with high adhesion rate; but only suitable for cartilage defect in some specific sites
MSS	M-cells were cultured on the surface of the scaffolds, and then the scaffolds were put into a MF for few hours	Increase the number of cells attached to the scaffold; enhance infiltration and distribution; enhance Col II and aggrecan expression
Magnetic scaffold	MNPs are cross-linked with extracellular matrix materials, and then an external MF was applied during forming the scaffold structure	Program aligned nanofibers with several degrees of anisotropy in scaffold; support the formation of a biologically cell layer that protect the material; have superior structure and mechanical performance
MagCSs	Based on the cell sheet technology, m-cells exposed to MF assemble into sheet-like structures to stack layer-by-layer	Cell monolayer could be assembled layer-by-layer and still keep an intact cell sheet; contain different cocultured cell types; scaffold free

*MAM, magnetically actuated manipulation; MF, magnetic field; MTD, magnetic targeting delivery; MSS, magnetic force-based cell seeding into the scaffolds; MagCSs, magnetic cell sheets; m-cells, magnetic labeling of cells; MNPs, magnetic nanoparticles.*

### Guiding Cells to a Targeted Defect Site

Precise guidance of individual cells have great potential to provide convenient cellular microenvironment (Guillotin and Guillemot, 2011). Cells with magnetic force provides minimized interference with the biochemical functions of cells. We defined this procedure as magnetic targeting delivery (MTD). This technique avoided excessive number of MSCs that generated free bodies of scar tissue (Agung et al., 2006). In stead, a relatively small number of MSCs were enough to accumulate to a desired area (Hori et al., 2011; Kamei et al., 2013). Mahmoud et al. (2016) indicated that 2 × 10^5^ m-MSCs were suitable to attain complete repair of the cartilage repair (3 mm in diameter and 4 mm in depth). Unlike intra-articular magnet that needs to be removed after translation, the external magnetic force system with less invasiveness is easy to handle, and could be completed under arthroscopy. In addition, m-MSCs exposed to MF could adhere to the target site physically, and then upregulate the expression of adhesion proteins such as integrin α2, integrin α6 and integrin β3 (Nakamae et al., 2010). Even removing the external MF, m-MSCs could still remain at the defect site (Hori et al., 2011). After the MF exposure, m-MSCs have a good adhesion, like cell sheets covering to the defect surface, which can favor cell communication and signaling among cells that are capable of spontaneous matrix formation and further promote chondrogenesis and cartilage regeneration (Goncalves et al., 2017; Vidiasheva et al., 2018). Thus, MTD could make a potential homogeneous distribution of m-MSCs to cover the defect area with high adhesion rate, which have trended toward promoted chondrogenic differentiation and cartilage regeneration. However, the application of MTD was only suitable for cartilage defect in some specific sites, such as the center of the patella and the center of lateral or medial tibial plateau, due to the fact that magnetic force is generated in only one direction and magnetic force becomes weak far from the surface of magnetic device (Kamei et al., 2013). Besides, with the friction of knee motion, there existed a risk of attached cells peeling from the defect site after MTD.

### Enhancing the Seeding Efficiency of Cells Into the Scaffold

Obviously, cells and scaffolds are the essential parts of tissue engineering. The complex structure of scaffolds would cause difficulties in cell seeding, which resulted in a non-uniform and inadequate migration of cells into the scaffold (Melchels et al., 2010). It is well established that cell compaction was a critical step for cell differentiation, and the use of scaffolds could provide a suitable environment for cell proliferation and differentiation. However, a small number of MSCs seeding and lack of cell condensation into scaffold restricted chondrogenic differentiation, but another application of MAM in cell culture is magnetic force-based cell seeding into the scaffolds (MSS), which could enhance seeding efficacies to reach a sufficient cell density to promote cell condensation (Sensenig et al., 2012; Luciani et al., 2016). One study reported that magnetic labeled cells remained within pores of the scaffolds were tightly aggregated with magnetic confinement, while only individual cells were observed further from the magnetic (Luciani et al., 2016). Furthermore, MSS not only increased the number of cell attached to the scaffold, but also enhanced infiltration and distribution (Thevenot et al., 2008). Besides, MSS could enhance the rate and extent of Col II and aggrecan expression, which was favorable to chondrogenic differentiation (Fayol et al., 2013; Brady et al., 2017; Su et al., 2017). Considering the fact that the use of MTD is limited to some specific site of the knee joint, MSS seems to be more appropriate for the treatment of larger and full-thickness lesions without limitations of cartilage defect sites. However, the procedure of MSS needs surgical opening of the knee joint, which is more complicated and invasive than MTD.

### Formation of Magnetic Scaffolds

MAM was also used to assemble a 3D culture structure. MNPs are cross-linked with several extracellular matrix (ECM) materials, such as alginate solution, polyvinyl alcohol, hydrogel and other ECM proteins, and then an external magnetic field was applied during forming the scaffold structure. The scaffold structure can be modulated by an alteration in the MNPs concentration and the direction and intensity of magnetic fields to mimic the native cartilage ECM structure (Margolis et al., 2018). The magnetically guided assembled scaffold, without changing the bulk stiffness, could program aligned nanofibers with several degrees of anisotropy in scaffold structure and synthesized relevant ECM molecules, which would affect seeded cells and further development (Kim and Tanner, 2016; Margolis et al., 2018). Besides, the magnetic hydrogel, without any toxic or infectious agents, was able to capture and retain magnetic-labeled cells, maintain cell phenotype and function, delivery biomaterials of therapeutic agents and cells via minimally invasive procedures, and can be used to obtain high-density cell coverage on the defect sites for cartilage repair (Bonnier et al., 2015; Xu et al., 2019). Moreover, it could support the formation of a biologically cell layer that protect the material from inflammation (Arias et al., 2018). And Wong et al. (2017) demonstrated a monolayer of RGD-bearing magnetic nanoparticles to tune the tether mobility of RGD on substrate by active mechanotransduction signaling via magnetic force, and found that MSCs cultured on this magnetic scaffold have better adhesion, spreading and osteogenic differentiation. In addition, magnetic scaffolds, with superior structure and mechanical performance, are conductive to cell adhesion and proliferation (Zhao et al., 2019). If used more effectively, magnetic scaffold with seeded cells could stimulate chondrocyte-related gene expression (Huang et al., 2018), which could be further used in cartilage repair. However, the procedure of magnetic scaffold is more complicated than MTD and MSS.

### Guiding Cells Assembly Into Sheet-Like Structures to Stack Layer-by-Layer

Current 3D cell culture methods are dependent on biocompatible scaffolds, which require complex syntheses and fabrication steps. Cell sheet technology can delivery cells in an ECM context to guide the cells upon implantation without any scaffolds. Magnetic-labeled cells could be aggregated into spheroids at a targeted location on the attachment surface to form 3D arrangements in a convenient microenvironment via MAM which is an available procedure to mimic tissue properties of cells (Souza et al., 2010; Ghosh et al., 2016). Additionally, MNPs exposed to MF had the ability to make m-cells cohere together in order to reproduce different 3D geometries and cellular compositions (Souza et al., 2010). Furthermore, the layers of cell sheets could be brought into close proximity by magnetic force (Koto et al., 2017). Thus, magnetic cell sheets (MagCSs) can be used for the formation of scaffold-free 3D cell culture and cellular assemble.

The MagCSs, largely maintained by the formation of cell-to-cell junctions and secretion of ECM proteins, are free from the restriction of scaffolds, which could avoid the adverse effects of scaffolds (Gil et al., 2015). In MagCSs, the cell monolayer could be detached spontaneously via magnetic levitation and still keep an intact cell sheet. In addition, the shape of cell sheets could be precisely controlled via different magnet patterns, and it could also be manipulated to facilitate magnetized cell sheets transfer and scaffold-free tissue formation through the application of magnets (Penland et al., 2017). With the application of magnetic technology, the cell monolayer could be precisely controlled to stack layer-by-layer to form multilayer sheets or containing different cocultured cell types, which cannot be achieved by classical method of cell-sheet preparation (Ito et al., 2007; Zhang et al., 2017). Additionly, the MagCSs had a potential of angiogenesis in ischemic tissues that were attributable to an increased expression of vascular endothelial growth factor and reduced apoptosis in ischemic tissues (Ishii et al., 2011). Besides, Zhang et al. (2017) reported that multilayered cell sheets incorporated with growth factors via MAM could induce bone formation. Furthermore, they also successfully constructed a composite tissue (two-layer chondrogenic and osteogenic cell sheets) using MAM to mimic an integrated osteochondral complex with a cartilage-bone junction, and cells in respective sheets were observed to differentiate into chondrocytes and osteoblasts, respectively. If used more effectively, MagCSs, free from the restriction of scaffolds, could match the thickness and the shape of cartilage lesion, and mimic the natural composition of cartilage tissue, which could be further used in cartilage repair.

## The Effects of MAM *In Vivo* in Cartilage Regeneration

The first detailed *in vivo* study of MAM was reported by Kobayashi et al. (2008), who evaluated its efficacy and safety in animal models with osteochondral defects. They injected 5 × 10^6^ m-MSCs and accumulated to the cartilage defect with an external magnetic force or 10 min. The process of accumulation were observed via arthroscopic evaluation. At 12 weeks after surgery, the arthroscopic scores and histological scores (Wakitani scoring system) were significantly better in the MAM group than in the MSC group and control group. Besides, Kamei et al. (2013) reported the efficacy of MTD in 16 mini-pig cartilage defect models, and found that the adhesion rate of m-MSCs transplanted to the cartilage defect site was increased by MTD, compared to the case treated with the gravity adhesion technique. Moreover, transplanted m-MSCs still retained their chondrogenic potential. And Mahmoud et al. (2016) who evaluated 26 white rabbits treated with MTD showed efficient cartilage repair even in a case of severe chronic osteochondral defect. In addition, Hori et al. (2011) demonstrated that transplant magnetic-synovium-derived cells (m-SDCs) to the defect site have chondrogenic potential with thick layers of chondrocyte-like cells in the defects and better Wakitani scale and modified O’Driscoll scale at 12 weeks after surgery. And Kotaka et al. (2017) transplanted the magnetically labeled pluripotent stem cells (m-iPS) into the osteochondral defect site of a nude rate in the presence of MF, and indicated that MTD of m-iPS could improve the cartilage regeneration. For safety evaluation after injection of m-MSCs into the knee joint, assessments of *in vivo* kinetics of transplanted m-MSCs revealed that m-MSCs were not present in any major organs (brain, heart, lungs, liver, kidneys, spleen) after intraarticular administration, regardless of magnetic targeting or not (Ikuta et al., 2015).

## The Application of MAM in Clinical Trial

However, only one study reported MAM applicated in clinical cartilage repair. Kamei et al. (2018) evaluated five patients treated with MAM at 48 weeks follow-up. The average defect size was 2 cm^2^ area. Exposing to a 1.0 T MF, 1 × 10^7^ m-MSCs containing in 5 ml of saline were injected into the knee joint. After 48 weeks follow-up, their data showed that all five patients were satisfied with the clinical outcomes, reporting a significant improvement of IKDC and KOOS score. MRI and arthroscopy finding showed complete coverage of the defect sites in three patients. Swelling of treated knee joint occurred in three patients, two of who resolved within 2 weeks, but one still remained at 48 weeks. No other adverse events were observed during treatment.

## Conclusion and Further Perspectives

Benefiting from the advances in magnetically actuated manipulation for cartilage regeneration has attracted increasing interests. The current state-of-art research indicating the great potential of magnetic strategies for cartilage tissue engineering. Different strategies of magnetically actuated manipulation were summarized into the following aspects: MTD could deliver m-cells to the targeted cartilage defect sites with high adhesion rate; MSS could condense m-cells into the scaffold with magnetic confinement; magnetic scaffold could provide superior structure and mechanical performance for cell adhesion and proliferation; and MagCSs, with multilayer cell sheets or containing different cell types, could match the thickness and the shape of cartilage lesion without any restriction of scaffolds.

However, there are still several challenges to be addressed before its further application. In future, it should answer the following questions: what is the influence of dispersed or localized form of MNPs in conjunction with a MF on the intracellular modulation; what is the mechanisms of chondrogenic differentiation after cell targeting, magnetic seeding or MagCSs formation, whether it has similar outcomes as those of ACI or other one-step procedures, and whether it could relieve pain and shorten the rehabilitation process. To date, *in vivo* studies reveal the potential of MAM to stimulate chondrocyte-related gene expression and to promote chondrocyte-like cells regeneration. However, the ability to mimic or regenerate native extracellular matrix, which is essential for cartilage repair, should be further researched.

## Author Contributions

CZ, Y-ZC, X-JL, and YW conceived the study. CZ and Y-ZC wrote the manuscript. All authors contributed to the article and approved the submitted version.

## Conflict of Interest

The authors declare that the research was conducted in the absence of any commercial or financial relationships that could be construed as a potential conflict of interest.
